# FUMEPOC: Early detection of chronic obstructive pulmonary disease in smokers

**DOI:** 10.1186/1471-2458-11-413

**Published:** 2011-05-31

**Authors:** Vicente Gil-Guillén, Domingo Orozco-Beltrán, Concepcion V Carratala Munuera, Carlos Plaza-Sirvent, Patricia Lorca-Amorrich, Adriana López-Pineda, María P Vela-Troncoso, Juan J Soler, Manuel Yarza-Cañellas, Antonio Fernández, Luis Rosado-Bretón, Carmen Olivares-Bautista, Alejandro Muñoz-Fernández, FUMEPOC Study Research Team

**Affiliations:** 1 Departamento Medicina Clínica, Universidad Miguel Hernández de Elche (Campus de San Juan), 03550 San Juan de Alicante, Alicante, Spain; 2 Unidad Investigación, Hospital Universitario San Juan de Alicante, Carretera Nacional 332 Alicante-Valencia s/n, 03550, Sant Joan d'Alacant (Alicante), Spain; 3 Departamento Medicina Clínica, Universidad Miguel Hernández de Elche, Carretera Nacional 332 Alicante-Valencia s/n, 03550, Sant Joan d'Alacant (Alicante), Spain; 4 Departamento de Medicina Interna, Hospital General de Elda (Alicante), Ctra. Sax-Elda, s/n, 03600, Elda, Spain; 5 Servicio de Neumología, Hospital de Requena, Casablanca, S/N, 46340, Requena, Spain; 6 Agencia Valenciana de Salud, Conselleria de Sanidad, Micer Mascó no 31, 46010, Valencia, Spain; 7 Centro de Salud Villena II, Avenida Los toreros s/n, 03400, Villena (Alicante), Spain; 8 Hospital General de Elda Virgen de la Salud, Ctra. de Sax s/n, 03600, Elda, Spain

## Abstract

**Background:**

Currently is not feasible using conventional spirometry as a screening method in Primary Care especially among smoking population to detect chronic obstructive pulmonary disease in early stages. Therefore, the FUMEPOC study protocol intends to analyze the validity and reliability of Vitalograph COPD-6 spirometer as simpler tool to aid screening and diagnosis of this disease in early stages in primary care surgery.

**Methods / Design:**

Study design: An observational, descriptive study of diagnostic tests, undertaken in Primary Care and Pneumology Outpatient Care Centre at San Juan Hospital and Elda Hospital. All smokers attending the primary care surgery and consent to participate in the study will undergo a test with Vitalograph COPD-6 spirometer. Subsequently, a conventional spirometry will be performed in the hospital and the results will be compared with those of the Vitalograph COPD-6 test.

**Discussion:**

It is difficult to use the spirometry as screening for early diagnose test in real conditions of primary care clinical practice. The use of a simpler tool, Vitalograph COPD-6 spirometer, can help in the early diagnose and therefore, it could improve the clinical management of the disease.

## Background

Chronic obstructive pulmonary disease (COPD) is defined as a preventable and treatable disease with some significant extrapulmonary effects that may contribute to the severity of symptoms in individual patients. Its pulmonary component is characterized by airflow limitation that is not fully reversible. The airflow limitation is usually progressive and associated with an abnormal inflammatory response of the lung to noxious particles and gases [1].

The association between tobacco and COPD is well known and the best option to prevent a high risk of suffering from COPD for these patients is to quit this smoking habit [2].

By means of epidemiological studies it has been quantified that COPD is a common problem and that the fact of being under-diagnosed and undertreated makes its prognosis worse [3-7]. COPD is usually classified in 4 stages according to its severity. The latest evidences in COPD have proven that early detection in stages 1 and specially in stage 2 achieve significant improvements in the natural history of the disease [8,9].

Spirometry is currently the standard assessment tool for diagnosing, staging and monitoring disease progression. It is considered the most objective and reproducible measurement of airflow limitation [1].

The ratio of the two measurements (FEV1 ⁄ FVC) is calculated to assess a patient's lung function. In patients with COPD, FEV1 and FVC readings, as well as FEV1 ⁄ FVC ratios, will be lower than predicted (reference) values based on age, sex, height and race. Airflow limitation is clinically confirmed when the FEV1 ⁄ FVC postbronchodilator value is < 0.70 [1].

Various types of spirometers may be used in assessing and monitoring COPD. But conventional spirometry is difficult to use in primary care because lack of time and need of specific training. Recently other types of spirometers have been introduced that are portable, readily available, and easy to use in primary-care settings. With lower needs of instruction and training in spirometry techniques, healthcare providers can easily incorporate spirometry into their practices and increase the likelihood of earlier detection of COPD among their patients [10]

Therefore, it was decided to develop this study in order to establish the validity and reliability of Vitalograph COPD-6 spirometer (COPD-6), and to determine the prevalence of undiagnosed COPD in smokers.

The COPD-6 identifies likely cases of COPD by measuring patients' obstructive index and FEV1/FEV6 ratio. Those whose measurements are within normal range can be screened out, allowing diagnostic spirometry resources to be focussed on those most at risk. A previous study about COPD shows acceptable validity indexes but another one shows low sensitivity [11,12].

## Methods / Design

### Main objective

To establish the validity and reliability of COPD-6 compared with conventional spirometry (CS).

### Specific objectives

1. To determine the prevalence of undiagnosed COPD in smokers.

2. To establish the saving times in the primary care setting by using COPD-6 before CS instead of CS only.

### Design

FUMEPOC is an observational descriptive study of diagnostic tests.

### Setting

The sample will be recruited from the province of Alicante (Spain) and, specifically, the population receiving healthcare from 5 primary care surgeries at Elda Health Department and San Juan Health Department. So 5 Health centres and 2 Hospitals were involved.

### Study Population

Patients 45 years of age and over who are smokers with a pack-year index greater than 10, without known respiratory disease attending a primary care consultation will be included (Table 1). Patients who refuse to perform spirometry or not giving in the informed consent were excluded.

**Table 1:** Criteria to include patients in the FUMEPOC study

Age 45 years or older
Smokers with pack-year index greater than 10

Free of known respiratory disease

Informed consent

Patients undergo a test with Vitalograph COPD-6 spirometer. Subsequently, a spirometry (Datospir-120) will be performed as the gold standard test at the reference hospital and the results will be compared. Patients with acute respiratory disease will make the both tests in a different day once the acute disease has disappeared (at least 6 weeks after acute disease).

For sample size calculating, after bibliography searching, high variability in sensitivity and specificity criteria was found. For that reason we assume the maximum uncertainty. Thus, the sample size is calculated by accepting a sensitivity or specificity = 50%. A 95% confidence level and a 5% precision are assumed. Because of the characteristics of the transversal design of the study, in which patients are screened by the regular family physician, and the absence of complex tests or high-risk tests, we suppose a low percent loss. Therefore we assume a 20% loss. According these assumptions, approximately 480 patients will be needed. Calculations are made with qualitative variables formula for infinite populations in the estimation of a validation parameter (sensitivity / specificity), subsequently the correction formula is applied.

### Methods of Data Collection

**Figure 1:**
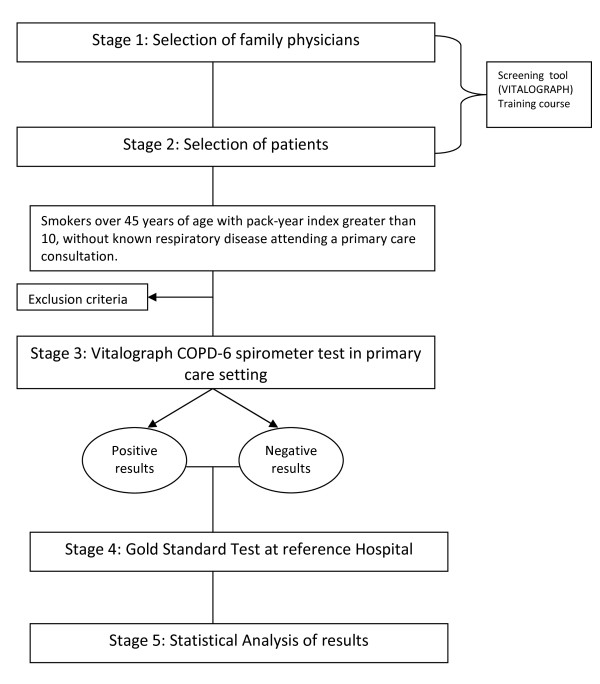
**Flow diagram of the FUMEPOC study**.

The number of primary care physicians (PCP) who will participate in the study should be about 40. Each PCP will be trained on the screening tool (COPD-6) and after that will select 10 consecutive patients from daily clinical practice who meet the inclusion criteria (Table 1). Once the patient is included, PCP applied the screening test and asks the patient about some related variables (Table 2). All included patients (screening positive or negative) will be referred to perform the gold standard test, CS, to be done at the reference Hospital of San Juan or Elda. Figure 1 provides a flow diagram of the study.

**Table 2:** Study variables in FUMEPOC study

Variables	Collection method
Socio-demographic	Clinical record

Smoking status	Clinical record

Pack-year index = (no cigarettes per day/20) × smoking years	Clinical record

Symptoms (morning cough, sputum) and others (specify)	Clinical record

Spirometer device COPD-6 (FEV 1, FEV 6 and FEV1/FEV6)	Device result

Evaluation of screening test results	Test

Personal history and cardiovascular diseases	Clinical record

Consultation Time (physician)	Timer/Chronometer (minutes)

Referral Time (patient)	Days

Cholesterol (HDL and LDL), PAS, PAD, BMI	Laboratory results taken from the clinical record

COPD will be diagnosed according to the most widely accepted criteria, that is, FEV1/FVC < 0.7 and a history of smoking habit. The severity of COPD will be determined according to the GOLD criteria: I (FEV1 > 80%, II (FEV1: 50-80%), III (FEV1: 30-49%); IV (FEV1 < 30%)

### Vitalograph COPD-6 spirometer. Interpretation of the results

The following results were considered: NORMAL (FEV1/FEV6 > 0,7), STAGE I: Mild (FEV1/FEV6 < 0,7 y FEV1 > 80%), STAGE II (FEV1/FEV6 < 0,7 y FEV1 < 80%), STAGE III (FEV1/FEV6 < 0,7 y FEV1 < 50%) and STAGE IV: Severe (FEV1/FEV6 < 0,7 y FEV1 < 30%).

### Measurements

Table 2 shows all the study variables and the collection method. Smoking status will be measured according to WHO's Smoking and tobacco Use Policy. A smoker is defined as someone who smokes any tobacco product, either daily or occasionally.

### Statistical Analysis

Univariate and bivariate analysis will be performed to describe the variables and to analyze validity indicators. For those related to the spyrometers' screening, the sensitivity, specificity, positive predictive value and negative predictive value (PPV and NPV) and likelihood ratio will be calculated; for the values of the diagnostic tests which follow a quantitative scale the ROC curve (receiver operating characteristic) will be used in order to facilitate the cut-off point.

Confidence interval is used at 95% for all variables.

### Ethical and Legal Issues

The FUMEPOC study protocol has been reviewed and approved by the Committee for Ethics and Clinical Trials of San Juan Hospital (*Comite Ético de Investigación Clínica (CEIC) de Hospital de San Juan*)

#### Legal Aspects

The study is conducted according to the standards of the International Guidelines for Ethical Review of Epidemiological Studies (Council for International Organizations of Medical Sciences- CIOMS-Geneva, 1991) and the recommendations of the Spanish Society of Epidemiology about the review of ethical aspects of epidemiological research.

##### Confidentiality of the data

All information relative to the patient's identity is considered confidential. The data generated during the study will be handled according to the Spanish Law 5/1999 and corresponding norms. All of the researchers will be required to sign a confidentiality agreement in order to access the study data.

##### Informed consent

*All patients must read the "Patient Information Form" and sign a document giving in consent*.

## Discussion

COPD is a leading cause of morbidity and mortality worldwide, and results in an economic and social burden that is both substantial and increasing. The prevalence and morbidity data greatly underestimate the total burden of COPD because the disease is usually not diagnosed until it is clinically apparent and moderately advanced [2].

Currently, COPD is a more costly disease than asthma and, depending on country 50-75% of the costs are for health services associated with exacerbations. Tobacco smoke is by far the most important risk factor for COPD worldwide. Other important risk factors are occupational exposures, socio-economic status and genetic predisposition [2].

COPD is the fourth-leading cause of death in the USA and Europe, and COPD mortality in females has more than doubled over the last 20 yrs [13].

This study is developed in the Health Departments of San Juan and Elda (Alicante, Spain), with enough research personnel and material resources for its implementation (spirometers-CS-, COPD-6 spirometers, clinical rooms, and others). Family doctors of these Departments will perform the study once a training course was made.

An early diagnosis of COPD is important, since depending on the stage in which it is made, different treatments or actions can be applied. This is important because currently there are no treatments that can reverse the natural history of disease in later stages [6,7].

Some limits of this diagnostic tests study are related to those from diagnostic observational studies for the validation of a measure device, such as diagnosed verification bias. It must be taken into account to consider the validity of the results.

In order to solve some kind of bias some measures will be taken. Both tests should be applied to all patients (positive and negative screening results) in order to obtain the diagnoses confirmation or not in all cases.

Patients were selected from Primary care setting by their family doctors so the percentage of non-responders is expected to be low avoiding selection bias.

This study includes only smokers because of the increased risk for COPD but in the future and depending on the results a second phase will be conducted with ex-smokers.

The "gold standard test" should be applied blindly to the evaluator that is not aware of the outcome of the screening test in order to avoid information bias. So that, the COPD6 result will be not included in the petition sheet of spirometry in the hospital and the result of screening will be not given to the patient until both tests have been performed. So a final report on the results of the two tests will be sent to patients.

Prior to the start of the study reproducibility of the test must be assessed. A poorly reproducible test may affect the validity of the results. In our study the screening instrument COPD-6, is a perfectly reproducible test in patients with different clinical and pathological states.

Regarding "gold standard" selection, Conventional Spirometry is the gold standard for diagnosing the disease and monitoring its progression as it is non-invasive, standardised, reproducible, and objective. Functional diagnosis of COPD is important for identifying and quantifying airflow limitation, reversibility, disease severity and exacerbations. Functional diagnosis is also important for long-term therapeutic monitoring and for establishing the need for pulmonary rehabilitation [14].

Spirometry ought to be used in primary care as a screening tool for the early detection of COPD in all patients > 45 years of age who are currently smoking, as well as those with respiratory symptoms [15].

But Spirometry is difficult to use widely in PC setting. To implement screening tools in primary care it needs to be trough a simpler, cheaper test because of the primary care clinic conditions [16].

Spirometry enables the primary care health professional to make an objective measurement of airflow limitation and the degree to which it is reversible, and is an important tool for accurate diagnosis and effective management of chronic respiratory diseases including asthma and COPD [17]. However, spirometry remains underused in primary care [18,19]. Barriers to performing spirometry in community settings include lack of access to calibrated spirometers, inadequate training in performing spirometry, price of conventional spirometers, lack of quality-control systems to ensure accurate results, and inadequate interpretation skills among health professionals performing the test [20,21].

Having a simple screening tool adapted to the primary care setting would detect COPD in early stages and the number of patients referred to CS will be lower. It could help to establish a protocol to facilitate high risk people screening with high rates of compliance. This is an effort in terms of prevention, which can result in fewer later stages cases of the disease and consequently, better quality of life for patients. In addition it could help savings for the healthcare burden.

## Abbreviations

COPD: Chronic obstructive pulmonary disease; PPV: Positive predictive value; NPV: Negative predictive value; ROC: Receiver operating characteristics; PCP: Primary care physicians; SBP: Systolic blood pressure; DBP: Diastolic blood pressure; BMI: Body mass index; CS: Conventional spirometry

## Competing interests

The authors declare that they have no competing interests.

## Authors' contributions

VG, DO and CVCM participated in the design of the study. VG, DO, CVCM, CP, PL, AL, and MPV contributed to the protocol development. CP, PL, AL, and MPV participated in the manuscript writing. JJS, MY, LR, CO, AM and AF contributed to the manuscript review.

All the authors have read the draft critically, to make contributions, and have approved the final text.

## Pre-publication history

The pre-publication history for this paper can be accessed here:

http://www.biomedcentral.com/1471-2458/11/413/prepub
